# Spontaneous remission in diffuse large cell lymphoma: a case report

**DOI:** 10.1186/s13256-018-1937-z

**Published:** 2019-02-01

**Authors:** J. Snijder, N. Mihyawi, A. Frolov, A. Ewton, G. Rivero

**Affiliations:** 1 Baylor St Luke Medical Center, Section of Hematology/Oncology, 1 Baylor Plaza, Houston, TX 77030 USA; 2 Department of Bioinformatics and Statistics, Baylor College of Medicine, Houston, TX 77030 USA; 3 Department of Pathology, Texas Methodist Hospital, Houston, TX 77030 USA; 4 The Dan L. Duncan Comprehensive Cancer Center at Baylor College of Medicine, Houston, TX 77030 USA

**Keywords:** Diffuse large B-cell lymphoma, Spontaneous regression, Ageing, Inflammation

## Abstract

**Background:**

Spontaneous remission in solid malignancies has been documented. However, spontaneous remission in aggressive diffuse large b cell lymphoma is exceedingly rare. Previous reports of lymphoma remission suggest that not yet fully characterized tumor-intrinsic and microenvironment mechanisms cooperate with spontaneous regression.

**Case description:**

Here, we report the case of an 88-year-old white woman with diffuse large b cell lymphoma (follicular lymphoma transformed) who achieved morphologic spontaneous remission 3 months after her diagnostic core biopsy. We examined 16 similar cases of diffuse large b cell lymphoma suggesting that spontaneous remission is preferentially observed in elderly patients soon after their biopsy microtrauma, especially if malignancies are Epstein–Barr virus driven and activated B-cell type.

**Conclusion:**

Our case and reported analysis highlight that anti-tumor adaptive T cell responses are potentially augmented in a subset of patients leading to lymphoma regression. In these patients, it is possible that “primed” innate anti-tumor T cell immunity is enhanced in immunogenic lymphoma subtypes after tissue biopsy. Our case and analysis not only reinforce the role of innate T cell anticancer immunity, but also originates potential proof of concept for investigation of unexplored pathways that could favorably impact T cell therapy.

## Introduction

Diffuse large b cell lymphoma (DLBCL) is an immunologic and genetic heterogeneous disease. Anthracycline-based chemotherapies combined with rituximab, such as the regimen of rituximab, cyclophosphamide, doxorubicin, vincristine, and prednisolone (R-CHOP) are normally administered for therapy [1]. However, spontaneous remission (SR) rarely occurs [2]. SR is defined as the complete or partial resolution of the tumor without administration of immunochemotherapy. SR is not exclusively observed in lymphomas and has been increasingly described in various malignancies, such as leukemia, malignant melanoma, Kaposi sarcoma, and neuroblastoma [3]. Although the mechanisms leading to SR remain uncharacterized, its infrequent occurrence highlights the possibility that tumor-associated molecular and patient-induced immunologic mechanisms cooperate to induce tumor regression. Previously, it has been hypothesized that a “favorable adaptive immunity” against concurrent bacterial or viral infection and even post biopsy trauma could render an “enhanced anti-tumor effect.” Understanding mechanisms leading to SR could improve cellular and immunotherapy challenges for treatment of lymphoma. In this study, we present an elderly patient diagnosed with DLBCL who experienced SR. In addition, to gain insight into the histologic, immunophenotypic, cytogenetic, and molecular features of patients experiencing SR, we examined 16 additional cases reported in the English literature. We found that advanced age, limited stage, and activated B-cell (ABC) phenotype associated with Epstein–Barr virus (EBV) are potentially linked with a subgroup of patients with propensity for SR.

## Case presentation

**Fig. 1:**
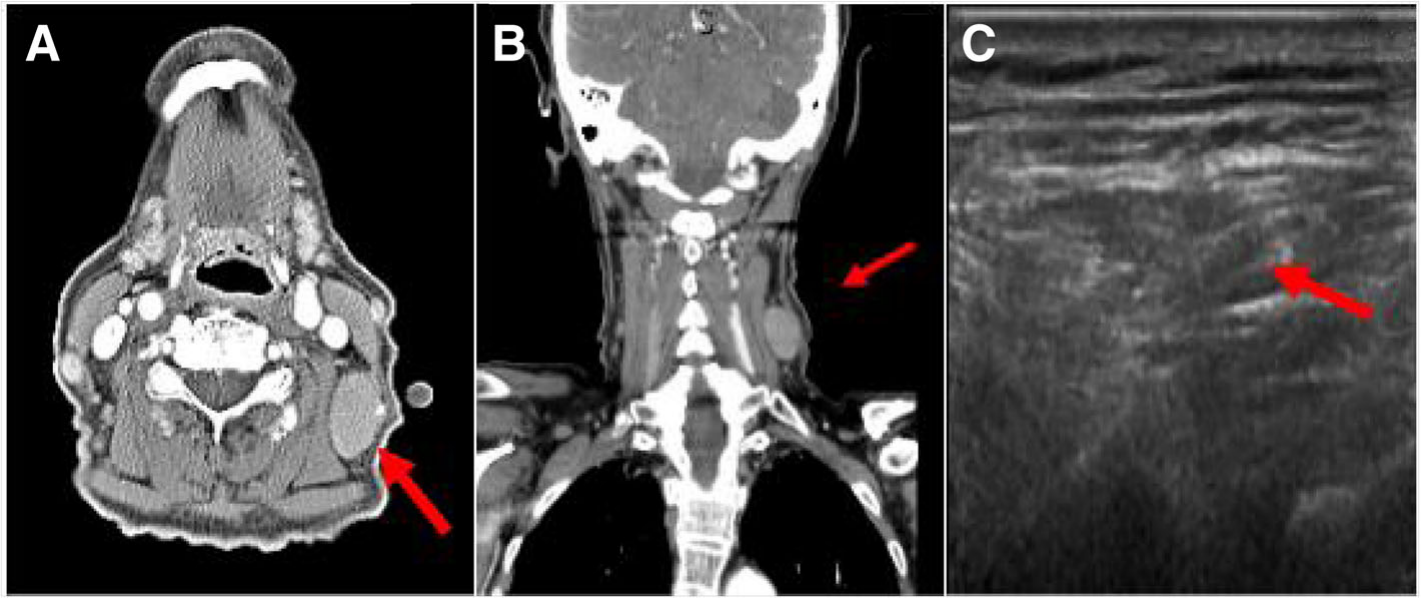
Initial neck computed tomography for cervical mass and follow-up neck ultrasound. **a** and **b** Neck computed tomography showing 4.5 cm level III left-sided neck mass (red arrows). **c** Neck ultrasound showing resolution of previously observed neck mass. Small non-pathologic appearing nodes with fatty hila were identified (red arrow)

**Fig. 2:**
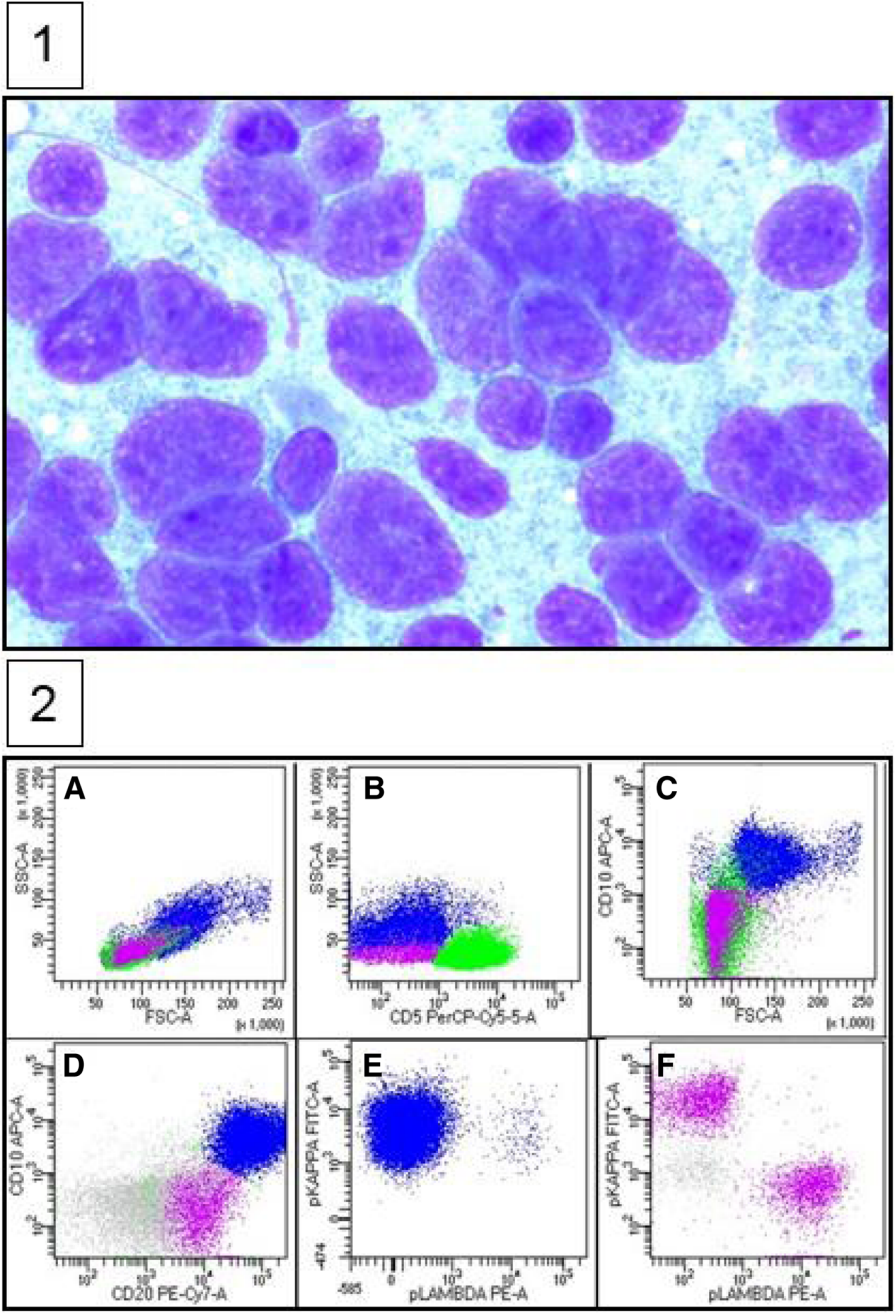
Lymphoma biopsy. **1** Cells are composed predominantly of large lymphocytes two to three times larger than normal background small lymphocytes (Diff-Quik stain, × 100). **2 a–f** Flow cytometry, left-sided neck mass: Flow cytometry of the left-sided neck mass shows a CD10 positive kappa light chain-restricted large B cell lymphoma with a background of small T cells and polytypic non-germinal center B cells. **a** Small T cells (*green*, 36% of cellularity) and small CD10 negative B cells (*purple*, 10%) with low forward scatter and large CD10 positive B cells (*blue*, 39%) with higher forward scatter. **b** CD5 positive T cells (*green*) and CD5 negative B cells (*purple* and *blue*). **c** CD10 positive B cells are larger by forward scatter than the T cells (*green*) and CD10 negative B cells (*purple*). **d** CD20 and CD10 positive large B cell lymphoma, germinal center type (*blue*) and CD10 negative B cells (*purple*). **e** CD10 positive B cells (*blue*) show clonal kappa light chain restriction. **f** CD10 negative B cells (*purple*) show polytypic light chain expression

**Fig. 3:**
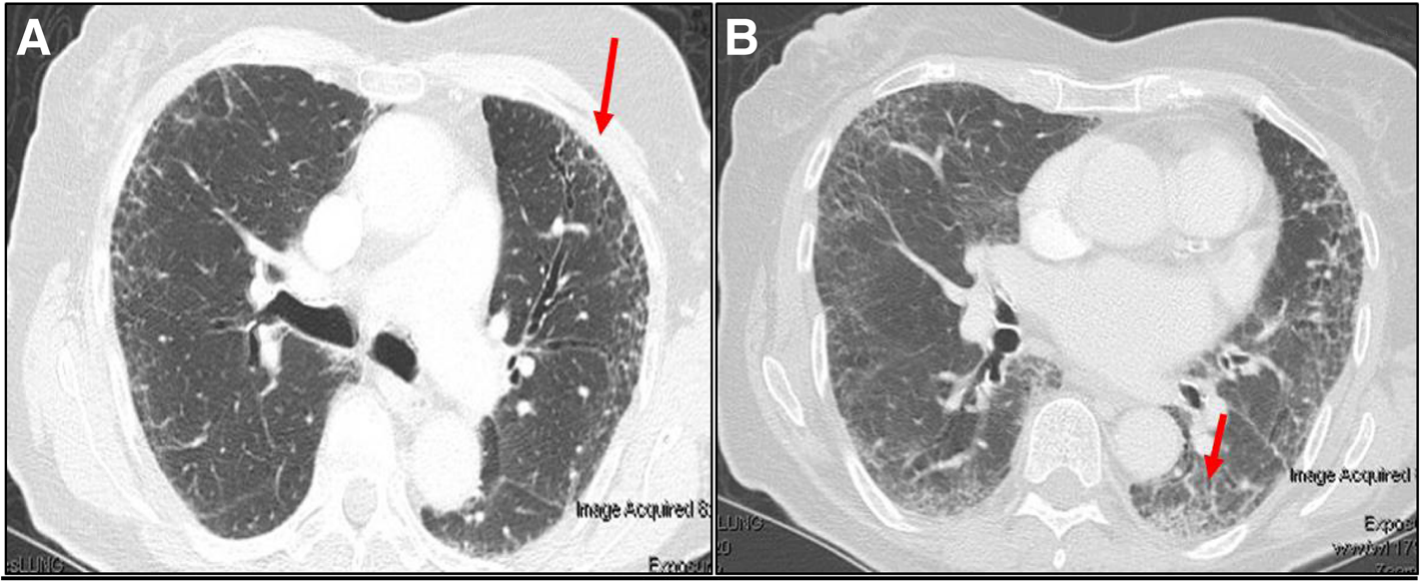
**a** Computed tomography demonstrates honeycombing (red arrow) distributed in the left lower lobe. Bronchial thickening is observed in the left lung. **b** Chest CT revealing subpleural ground glass opacities (red arrow)

**Fig. 4:**
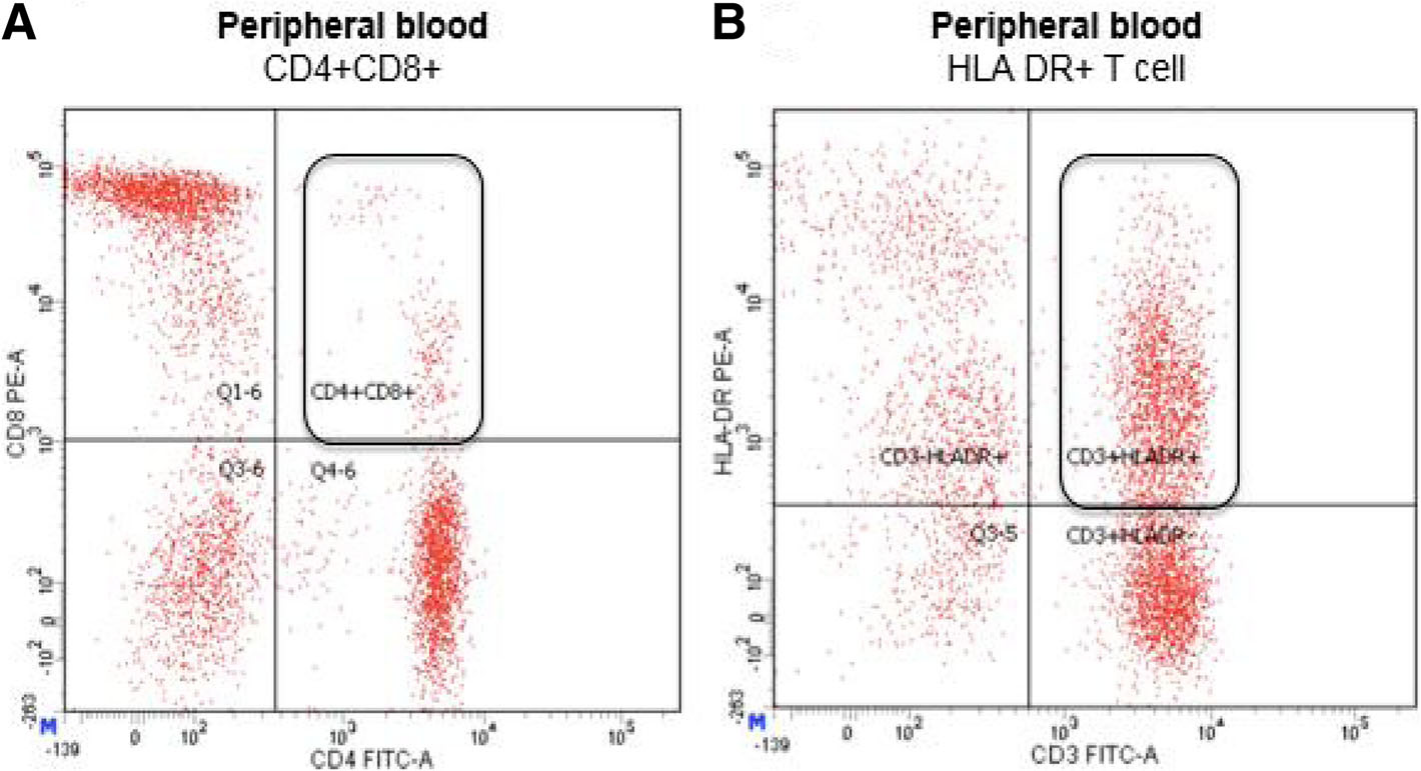
**a** Peripheral blood flow cytometry showing increased double-positive T-cells (DPT) in a patient exhibiting spontaneous remission of diffuse large B-cell lymphoma. **b** Peripheral blood flow cytometry T cell subtraction associated with increased HLA-DR. DPT and increased HLA-DR expression are linked to T cell activation and potential for cytotoxicity

An 88-year-old white woman with a history of vascular dementia and idiopathic pulmonary fibrosis (IPF) presented with a 4.5 cm left-sided level III anterior cervical lymph node (Fig. 1a and b). Prior to her onset of dementia and IPF, she was otherwise healthy. Her family history was not relevant for hematologic malignancies or cancer. She denied tobacco smoking. In addition to her neck mass, she developed night sweats and 1.8 kg (4 pound) weight loss. No lymph nodes were detected in her right supraclavicular, axillary, and inguinal regions. Auscultation of her lung bases revealed dry crackles. Hepatomegaly and splenomegaly were not observed. A computed tomography (CT)-guided core needle biopsy was done on September 16, 2014. A core, rather than excisional biopsy was considered given her severe lung disease and inability to tolerate general anesthesia. Tissue examination showed B cells of follicular origin, admixed with high-grade large cells (Fig. 2a and b). Flow cytometry showed clonal B cell population (36% of total cellularity) positive for CD10, CD19, and CD20 (Fig. 2b). Cells were Kappa-restricted associated with < 1% natural killer (NK) cells. Examination of her tumor biopsy showed a CD4:CD8 ratio of 9:1 without aberrant T cell antigen expression. EBV *in situ* hybridization was not performed. Positron emission tomography (PET-CT) revealed single uptake above the clavicle on the left side with standardized uptake value (SUV) of 4.9 confirming stage 1B disease. Her International Prognostic Index (IPI) was 2 points: low intermediate risk group; age > 60 years, lactate dehydrogenase (LDH) of 599, stage 1, Eastern Cooperative Oncology Group (ECOG) of 0. In addition, given her history of dry coughing and shortness of breath, a chest CT was obtained, which revealed honeycombing, bronchial wall thickening, and subpleural ground glass opacities suggesting interstitial pneumonitis (Fig. 3a and b). Her symptoms were controlled with inhaled β-agonists without administration of orally administered or systemic steroids. Her peripheral blood flow cytometry detected increased proportion of cells with cytotoxic potential including human leukocyte antigen-antigen D related (HLA-DR) + T cells (57%, normal 9–36%; absolute count of 884/mm^3^, normal 177–692/mm^3^) and double positive (DP) CD4/CD8 T cells (4%, normal 0–2%; absolute count of 62 mm^3^, normal 0–50 mm^3^) (Fig. 4a and b). After discussion of treatment options, she opted for best supportive care. Three months later, during a routine follow-up examination, it was noted that the lymph node had completely regressed. Ultrasonographic (Fig. 1c) and clinical remission were documented in October 2016, 25 months after her initial CT-guided biopsy. She died with progressive respiratory insufficiency attributed to IPF without evidence of lymphoma in December 2016.

## Methods

In addition, to this case report, 24 similar cases identified from the English literature were screened, which resulted in the selection of 16 representative cases for analysis [2, 4–17]. For all cases, high-grade large cell lymphoma confirmation without previous evidence for HIV infection, drug-induced immunosuppression, and a post-diagnostic procedure period that allowed observation without administration of cancer chemoimmunotherapy were required for selection. We investigated each patient’s demographics, tumor Ann Arbor staging, immunophenotypic staining for CD10, MUM-1, BCL-6, BCL-2, detectable possible infection (that is, EBV and *Helicobacter pylori*), time to SR, duration of response, remission status at the time of publication. Time to SR was defined as the time-period from biopsy for disease confirmation to complete/partial clinical or radiologic spontaneous tumor resolution. Our primary aim was to identify potential clinical, immunophenotypic, and molecular variables that could facilitate spontaneous tumor regression.

## Cohort analysis

The median age of the studied cohort was 68 years (range, 2–94 years) (Table 1). Four of the reported cases (23.5%) had disease limited to lymph nodes. Extranodal sites including leg, gastric, and breast were commonly observed. Stage I was observed in 12/17 (70.5%), whereas stage II and III were observed in 2/17 (11.7%) and 3/17 (17.6%), respectively. The median age for patients with stage I versus more advanced stage (II to IV) was 66.5 years (range, 2–88) versus 85 years (range, 82–94), *p* = 0.0008. A cell of origin (COO) by Han’s algorithm [18] was predicted based on immunophenotypic data available in 9/17 (53%) of cases. ABC phenotype was suggested in 8/9 (89%) of cases. The presence of EBV and *H. pylori* were evaluated by different techniques including *in situ* hybridization for EBV-encoded small RNA (EBER), immunohistochemistry for EBV-latent membrane protein 1 (LMP1) and for *H. pylori*, and Warthin–Starry staining, culture and serology. EBV was detected in 5/14 (36%) lymphoma samples and serology for *H. pylori* was positive in 3/3 (100%) of gastric DLBCL. Despite large cell histology documented in gastric tumor, eradication was unexpectedly achieved after *H. pylori*-directed therapy. The median time to SR was 40 days (range 15–240 days). An interesting observation was a non-statistical trend for potential association between detectable EBV and faster time to SR: 40 days versus 75 days for EBV (+) versus EBV (−) status, *p* = 0.07. In our studied cohort, SR was sustained for a median of 18 months (range, 3–30 months). For patients with more advanced stage (stage II and III, 1 and 3 patients each), median SR duration was 7 months (range, 3–31) versus 23.5 months (range, 6–84) for those with stage I, *p* = 0.18. In the selected case reports, four deaths were documented in a subgroup of patients with more advanced age (median 87 years, range 82–94).

**Table 1:** Previous publications demonstrating diffuse large B-cell lymphoma spontaneous remission

Case	Age/sex	Site	Size	Staging	CD10	MUM-1^a^	B C L-6^b^	BCL-2^c^	COO^d^	Additional findings	Time to SR^e^ (days)	Type of response	Additional treatment	Response duration (months)	Outcome	Ref
1	88/F	LN × 1 Neck	4 cm	I	+	NA	NA	NA	NA	NA	105	CR	None	21	Remission	Case
2	72/F	Leg	3 cm	I	–	+	NA	+	ABC^f^	EBV-^h^ (intra-tumor CD3, CD4 and CD8 ^+^ cells observed)	NA ^r^	PR^j^	None	21	Remission	4
3	66/F	Tongue	2 × 1 cm	I	–	+	–	+	ABC	EBV −	180	CR^k^	None	84	Remission	5
4	71/F	Breast	2.6 cm	I	–	+	+	+	ABC	EBV −	30	CR	None	18	Remission	6
5	2/M	Mastoid	Tumor fills sinus cavity	I	–	NA	–	+	ABC	EBV +	40	CR	None	24	Remission	7
6	60/F	Gastric	1–2 cm	I	NA	NA	NA	–	NA	*H. pylori* + ^i^ EBV −	139	CR	Anti-*H. pylori* ^m^	15	Remission	8
7	61/M	Gastric	2 cm	I	NA	NA	NA	–	NA	*H. pylori* + EBV −	75	CR	Roxatidine	6	Remission	8
8	73/F	Gastric	NA	I	NA	NA	NA	NA	NA	*H. pylori* +	40	CR	Anti-*H. pylori* ^n^	30	Remission	9
9	67/F	Max. sinus	5.1 × 4.2 cm	I	+	+	+	+	ABC ^p^	EBV −	240	CR	None	12	Remission	10
10	61/F	Spleen	Tumor diffusely infiltrated spleen	I	–	+		+	ABC	EBV +	15	CR	Prednisone ^o^	27	Patient relapsed requiring chemotherapy 27 months after diagnosis	2
11	40/F	Orbit/Conjunctiva	NA	I	NA	NA	NA	NA	NA	EBV +	40	CR	None	6	Remission	11
12	70/M	Jaw		I	NA	NA	NA	NA	NA	NA	20	CR	None	18	Remission	12
13	82/F	Leg	Multiple 2 cm lesions	II	–	+	+	+	ABC	EBV −	30	CR	None	4	(Stroke)✝	13
14	85/M	LN × 3.	0.5–2 cm	II	NA	NA	NA	+	NA	EBV −	30	CR	None	3	(Pneumonia)✝	14
15	94/F	LN × 3	1–2 cm	III	NA	NA	NA	NA	NA	EBV +	90	CR	None	10	(Pneumonia)✝	15
16	89/M	LN × 2	4 cm	III-IV	–	+	–	NA	ABC	EBV +	60	CR	None	20	(Pneumonia)✝	16
17	85/M	Prostate	Tumor diffusely involved prostate	III-IV	NA	NA	NA	NA	NA	NA	NA	CR	None	31	CR	17

^a^*MUM-1* multiple myeloma-1, ^b^ *BCL-6* B cell lymphoma-6, ^c^ *BCL-2* B cell lymphoma-2, ^d^ *COO* cell of origin, ^e^ *SR* spontaneous remission, ^f^ *ABC* activated B-cell, ^h^ *EBV* Epstein–Barr virus, ^i^ H. pylori *Helicobacter pylori*, ^j^ *PR* partial remission, ^k^ *CR* complete remission; ^l^, ^m^ *anti-*H. pylori *therapy* 1 week of lansoprazole/amoxicillin/clarithromycin, ^n^ *anti-*H. pylori *therapy* 6 weeks of lansoprazole/amoxicillin/clarithromycin, ^o^ *prednisone* patient received steroid therapy for hemophagocytic syndrome (HPS) at 15 months and 21 months without evidence of lymphoma recurrence, ^p^ suspected COO ABC given MUM-1 and BCL-6 positive status, *F* female, *LN* lymph node, *M* male, ^r^ *NA* not available,*✝* deceased

## Conclusions

In our study, we report the case of a patient with high-grade follicular lymphoma with high-grade transformation who exhibited SR after diagnostic biopsy. Our case and analyzed data suggest that advanced age, potential ABC COO, detectable tumor-associated infections (that is, EBV and *H. pylori*), and possibly preexisting autoimmunity could compound propensity for SR. Identification of patients diagnosed with high-grade lymphoma who experience SR is challenging given uniform necessity for administration of intense chemoimmunotherapy. However, in a selected minority of patients, advanced age and severe comorbid conditions could afford observation, after diagnosis is obtained, given high treatment-related mortality. Cancer cells circumvent anti-tumor T cell responses by activating “escape” mechanisms. Activation of cytotoxic T-lymphocyte-associated protein 4 (CTL4), programmed cell death protein-1 (PD1), and its ligand PDL-1, in tumor cells, contribute to tumor proliferation. Although tumor regression is feasible by optimizing autologous T/NK cell responses, SR has been reported without cytotoxic or adoptive cancer immunotherapy in patients with lymphoid malignancies. Notably, a common feature observed in our studied cohort is that SR developed in 95% of cases of DLBCL after tissue biopsy. The link between trauma and SR remains uncharacterized. However, massive lymphocytic infiltration and enhanced immune recognition represent a potential mechanism for tumor control [19] and suggests that pro-inflammatory “state” within the tumor core originating after biopsy renders potential for SR in specific patient subgroups. Tumors ameliorate inflammatory and efficient tumor-specific cytotoxic T cell (CTL) responses by upregulating prostaglandin E_2_ (PGE2), a metabolic product derived from cyclooxygenase-2 (COX2) allowing immunosuppressive state [20]. Interestingly, at the time of our patient biopsy, she had been taking aspirin at 325 mg orally daily for progressive dementia. It is possible that particularly for our patient, PGE2 depletion by using cyclooxygenase (COX) inhibitors, such as aspirin and celecoxib, enhanced anti-tumor T cell effector capacity. The median age for our patient cohort was 68 years suggesting that aspirin, a commonly prescribed medication among elderly patients, could have improved CTL tumor eradication.

Our observation that most of SR occurred in younger patients with early stage (70%, stage I) highlights that SR could be more frequently observed in less immunocompromised hosts with low tumor volume. However, we report an elderly patient diagnosed as having follicular lymphoma exhibiting high-grade transformation with long clinical latency for progressive pneumonitis/pulmonary fibrosis who developed SR. Her high peripheral blood T cells frequencies with increased HLA-DR and CD4/CD8 DP are probably linked with underlying autoimmunity such as IPF in our reported case or immunosenescence associated with ageing. It has been reported that autoimmunity correlates not only with elderly thymic involution but also with increased T cell autoreactive frequencies, suggesting that a similar phenomenon could have occurred in most of our patients who were older than 60 years described in our cohort. The cytolytic potential for CD4/CD8 DP T cells has been demonstrated in patients with cutaneous T cell lymphoma [21]. In the same patient, the CD4/CD8 DP population retained major histocompatibility complex (MHC) class I restricted activity against autologous tumor cells [21]. Buckner and colleagues’ and Abe and colleagues’ studies reinforce the observation that SR is feasible in patients older than 60 years with non-Hodgkin lymphoma (NHL) [10, 16]. Interestingly, a large number of publications addressing SR in patients diagnosed as having DLBCL originate from Asian countries suggesting a possible differential ethnic propensity for spontaneous tumor regression.

The oncogenic potential for infectious microorganisms in lymphoproliferative disorders has been extensively investigated. However, the mechanistic link between infectious neo-peptides, effector tumor-infiltrating T/NK cells, and lymphoma SR are not fully characterized. In our examined cohort, isolation of *H. pylori* was restricted to gastric DLBCL, whereas EBV detection was commonly observed in extranodal DLBCL associated with ABC COO. Analysis of case reports suggests that after biopsy, the median time to SR was, albeit not statistically significant, shorter in patients with clonally integrated EBV lymphoma. It is known that viral infections can upregulate MHC I expression in infected cells, facilitating recognition via T cell receptor (TCR) or NK cells [22]. Furthermore, EBV-dependent NHL retains significant antigenicity given incorporation of EBV viral sequences within its genome, making them susceptible to immune recognition [22]. In addition, the augmentation of natural killing activity could also be induced by viral infection [6, 23]. The role of anti-tumor T cell response after acute infections has been described by Buckner *et al*. [10], who reported the case of a patient diagnosed as having limited stage ABC DLBCL involving her right maxilla who developed progressive resolution of her facial swelling after experiencing pneumonia and acute *Clostridium difficile* gastroenteritis. Intriguingly, the authors suggest a potential cross-reactivity of pathogen-specific T cells against tumor antigens resulting in durable remission.

There are limitations to our analysis. First, given the retrospectively nature of our report, it is possible that inaccurate immunophenotyping resulted in overestimation of ABC cases. Second, although autoreactive T cells could have originated from ageing and autoimmunity, lacking *in vitro* T cell functional assays restricts our ability to assign a direct anti-tumor role. Despite the above limitations, our study emphasizes the possibility of SR in DLBCL. It would be interesting to investigate whether autoreactive T cells in elderly patients with autoimmunity retain activity against lymphomatous cells, and whether specific clonal expansion is augmented by biopsy microtrauma or even severe concurrent infections.

## Abbreviations

ABC: activated B-cell
COO: Cell of origin
CT: Computed tomography
CTL: Cytotoxic T cell
DLBCL: Diffuse large b cell lymphoma
DP: Double positive
EBV: Epstein–Barr virus
HLA-DR: Human leukocyte antigen-antigen D related
IPF: Idiopathic pulmonary fibrosis
MHC: Major histocompatibility complex
NHL: Non-Hodgkin lymphoma
NK: Natural killer
PGE2: Prostaglandin E_2_
SR: Spontaneous remission
